# Head CT is of limited diagnostic value in critically ill patients who remain unresponsive after discontinuation of sedation

**DOI:** 10.1186/1471-2253-9-3

**Published:** 2009-05-07

**Authors:** Jay S Balachandran, Mairaj Jaleel, Manu Jain, Niraj Mahajan, Ravi Kalhan, Rajesh Balagani, Helen K Donnelly, Eugene Greenstein, Gökhan M Mutlu

**Affiliations:** 1 Division of Pulmonary and Critical Care Medicine, Harvard Medical School, Boston, MA, USA; 2 Division of Pulmonary and Critical Care Medicine, University of Wisconsin, Madison, WI, USA; 3 Pulmonary and Critical Care Medicine, Rush University Medical Center, Chicago, IL, USA; 4 Division of Pulmonary and Critical Care Medicine, Northwestern University Feinberg School of Medicine, Chicago, IL, USA; 5 Division of Cardiology Northwestern University Feinberg School of Medicine, Chicago, IL, USA

## Abstract

**Background:**

Prolonged sedation is common in mechanically ventilated patients and is associated with increased morbidity and mortality. We sought to determine the diagnostic value of head computed tomography (CT) in mechanically ventilated patients who remain unresponsive after discontinuation of sedation.

**Methods:**

A retrospective review of adult (age >18 years of age) patients consecutively admitted to the medical intensive care unit of a tertiary care medical center. Patients requiring mechanical ventilation for management of respiratory failure for longer than 72 hours were included in the study group. A group that did not have difficulty with awakening was included as a control.

**Results:**

The median time after sedation was discontinued until a head CT was performed was 2 days (interquartile range 1.375–2 days). Majority (80%) of patients underwent head CT evaluation within the first 48 hours after discontinuation of sedation. Head CT was non-diagnostic in all but one patient who had a small subarachnoid hemorrhage. Twenty-five patients (60%) had a normal head CT. Head CT findings did not alter the management of any of the patients. The control group was similar to the experimental group with respect to demographics, etiology of respiratory failure and type of sedation used. However, while 37% of subjects in the control group had daily interruption of sedation, only 19% in the patient group had daily interruption of sedation (p < 0.05).

**Conclusion:**

In patients on mechanical ventilation for at least 72 hours and who remain unresponsive after sedative discontinuation and with a non-focal neurologic examination, head CT is performed early and is of very limited diagnostic utility. Routine use of daily interruption of sedation is used in a minority of patients outside of a clinical trial setting though it may decrease the frequency of unresponsiveness from prolonged sedation and the need for head CT in patients mechanically ventilated for a prolonged period.

## Background

Sedation is frequently required for comfort in patients who require mechanical ventilation for management of respiratory failure, but it is associated with the common complication of delayed awakening. While daily interruption of sedatives has been suggested to reduce ventilator and intensive care unit (ICU) days by avoiding delays in patient awakening, this may not be easily accomplished in patients who require high levels of oxygen and positive end expiratory pressure to maintain normal oxygenation [1]. Thus, persistent unresponsiveness or delayed awakening after discontinuation of sedation is not an uncommon problem in ICU patients.

Delayed awakening not only holds up the discontinuation of mechanical ventilation and increases length of stay in the ICU but may also prompt physicians to embark on a medical work-up to evaluate the cause of continued unresponsiveness after discontinuation of sedatives. Head computed tomography (CT) scans and other neurologic work-up are often obtained to further evaluate these patients. However, obtaining a head CT requires transportation of critically ill patients out of ICU, which can be challenging due to increased morbidity and mortality related to the transport itself. Therefore, the benefits of diagnostic testing should be weighed against the risks of transport before the decision for transport is made [2].

We noticed that in our medical ICU, persistent unresponsiveness after discontinuation of sedation is common and head CTs are frequently obtained as the initial work-up of persistent unresponsiveness after discontinuation of sedation in this patient population. We also observed that the decision to obtain head CT is not based on exam findings including neurologic examination without much thought being put into whether it will provide additional information or not. Persistent unresponsiveness after discontinuation of sedation can be due to multiple causes including metabolic complications, ongoing sepsis, epilepsy, encephalitis, cerebral anoxia as well as stroke. Therefore, head CTs are often obtained to further evaluate these patients; however, the diagnostic value of head CTs has not been evaluated in this specific patient population.

The purpose of this study was to evaluate whether obtaining head CT were useful for further assessment and management of mechanically ventilated ICU patients who remain unresponsive after discontinuation of sedation and who have a non-focal neurologic exam.

## Methods

### Subjects and Study Design

This was a retrospective review of consecutive patients in a single tertiary care medical center between November 2003 and November 2004. The study was approved by the Institutional Review Board at Northwestern Memorial Hospital and Northwestern University. Medical records from all patients admitted to the medical ICU between November 2003 and November 2004 were screened for study entry. Informed consent was waived because waiver of informed consent would not adversely affect the rights and welfare of the subjects and no data that will allow identification of the subject were collected. Inclusion criteria included the need for mechanical ventilation for respiratory failure for longer than 72 hours and failure to wake up with a non-focal neurologic exam after discontinuation of sedation. Forty-two patients out of a total of 380 screened met the entry criteria and their charts were retrieved for data collection. The inclusion and exclusion criteria of the study are summarized in Appendix 1. We also collected data from a control group of consecutive patients with respiratory failure requiring mechanical ventilation for longer than 72 hours who did not undergo a head CT evaluation for failure to wake up during the initial 4 months of study period (November 1, 2003–February 29, 2004).

Richmond Agitation-Sedation Scale (RASS) was used to monitor the depth of sedation. Although the target was set at -3 [(Moderate sedation), movement or eye opening to voice (but no eye contact)] or -4 [(Deep sedation), no response to voice, but movement or eye opening to physical stimulation), all patients had RASS score of -5 [(Unarousable), no response to voice or physical stimulation] based on neurologic exam findings [3,4].

### Data collection

Data collected included patient demographics, etiology of respiratory failure, duration and type of sedation prior to discontinuation of sedation, coagulation profile, laboratory data including renal and liver function tests, neurologic work-up including head CT, magnetic resonance imaging (MRI) of head, electroencephalography (EEG) and lumbar puncture.

### Statistical analysis

Data for patients who required head CT and those who did not were summarized using means and standard deviations for continuous variables and frequency distributions for categorical variables. Age between the experimental and the control group was compared using an unpaired t-test. Differences in sex, race, respiratory failure etiology, and sedation variables between the two groups were compared by Chi-square analysis. Statistical analyses to evaluate the distribution of data, calculation of median and interquartile ranges were performed using GraphPad Prism (GraphPad Prism 4 Software, Inc., La Jolla, CA). Significance was drawn at α = 0.05.

## Results

Three hundred and eight patients were admitted to medical ICU with respiratory failure requiring mechanical ventilation for longer than 72 hours. Forty-two (14%) of these patients remained unresponsive for at least 48 hours after discontinuation of sedation and had no underlying neurologic conditions or abnormal neurologic exams. All of these patients underwent neurologic work-up starting with a head CT. For the control group, we examined 144 consecutive admissions within the first 4 months of the study period who required mechanical ventilation for longer than 72 hours. Of these, 102 patients awakened easily after discontinuation of sedation, did not require head CT and were included in final control group analysis. Table 1 summarizes patient and control group demographics. There was no difference between patient group (patients who required head CT) and control group (patients who did not require head CT) for any of the variables in the demographics.

**Table 1:** Patient Demographics

	**Patients who required head CT **(n = 42) n (%)	**Patients who did not require head CT **(n = 102) n (%)
**Age**	62.6 ± 13.0 years	60.9 ± 10.4 years
**Gender**		
Male	24 (57)	53 (52)
Female	18 (43)	49 (48)
**Race**		
Caucasian	19 (46)	49 (48)
African-American	8 (19)	24 (23)
Hispanic	8 (19)	16 (16)
Asian	7 (16)	13 (13)
**Etiology of respiratory failure**		
Pneumonia	25 (60)	51 (50)
Extrapulmonary sepsis	7 (16)	22 (21)
Heart failure	5 (12)	8 (8)
Post-procedure	2 (5)	6 (6)
Variceal bleeding	2 (5)	5 (5)
Connective tissue disease	1 (2)	5 (5)
Neuromuscular disease	0 (0)	4 (4)

The median time from discontinuation of sedation to obtaining head CT obtained was 2 days (interquartile range 1.375–2 days). Head CT was non-diagnostic in all but one patient who had a small subarachnoid hemorrhage. Twenty-five patients (60%) had normal head CT. (Table 2) Thirty-seven (90%) patients regained consciousness and became communicative after discontinuation of sedation. The rest of the patients (10%) required reinstitution of sedation for continued need for mechanical ventilation or worsened clinically and never regained consciousness. Among these patients, there was no significant association between failure to recover and the type, dose and duration of sedation. Median time to regaining consciousness after discontinuation of sedation was 4 days (interquartile range 3–5 days), which was 2 days after head CT was obtained.

**Table 2:** Head CT Findings

**Finding**	**Number **(n = 42) n (%)
Normal	25 (60)
Chronic diffuse atrophy	8 (19)
Chronic small vessel ischemic changes	6 (14)
Chronic cerebral infarct	2 (5)
Acute intracranial hemorrhage	1 (2)

The patient who had the only diagnostic head CT showing a small subarachnoid hemorrhage recovered from coma without focal neurologic deficits 5 days after head CT was obtained. In addition to head CT, five patients had a brain MRI, which did not show any acute events that could explain the unresponsiveness. MRI brain were performed only in patients who had severe thrombocytopenia (<20,000 cells/mm^3^). The interval between head CT and MRI brain was 1 day. EEG was obtained in 10 patients showing no seizure activity in any of the patients. The most common result noted in EEG was diffuse slowing. Two patients underwent lumbar puncture for evaluation of cerebrospinal fluid, which was normal.

Two-thirds of patients (27 patients, 64%) had thrombocytopenia (<100,000 cells/mm^3^), and elevated aPTT (>35%). Fifteen (36%) patients elevated INR (>1.5). Only one patient had severe thrombocytopenia (<20,000 cells/mm^3^) and this patient was the one who had subarachnoid hemorrhage. Elevated creatinine (>2 mg/dl) was observed in one-third of patients (16 patients, 38%). None of the patients had elevated ammonia levels. There was no correlation between the presence of hepatic or renal dysfunction and the time to regaining responsiveness after discontinuation of sedation.

All patients received continuous sedation and were sedated longer than 7 days. The median duration of sedation before discontinuation of sedation was 12 days (interquartile range 7–14 days). There was no correlation between the duration of sedation prior to discontinuation and the time to regaining responsiveness. All patients received continuous sedation with a benzodiazepine (Lorazepam or midazolam) with or without fentanyl. Only eight patients (19%) had daily interruption of sedation. (Table 3). Analysis of the variables in the sedation method showed a statistically significant difference only in the use of daily interruption of sedation between patients and control group. Daily interruption of sedation was used more frequently in the control patients who did not have difficulty waking up after discontinuation of sedation and therefore did not need a head CT compared to patients who required a head CT for persistent unresponsiveness (37% vs. 19%, p < 0.05).

**Table 3:** Sedation method

	**Patients who required head CT **(n = 42) n (%)	**Patients who did not require head CT **(n = 102) n (%)
**Daily interruption of sedation**		
Yes	8 (19)	38 (37)*
No	34 (81)	64 (63)*
**Type of sedation**		
Intermittent	0 (0)	6 (6)
Continuous	42 (100)	96 (94)
**Type of sedatives**		
Lorazepam only	3 (7)	8 (8)
Midazolam only	1 (2)	3 (3)
Lorazepam + Fentanyl	31 (74)	70 (68)
Midazolam + Fentanyl	7 (17)	14 (14)
Propofol	0 (0)	7 (7)

*p < 0.05.

Two patients developed transient hypoxemia (SpO_2 _<90%) lasting less than 15 minutes temporally related to transportation to radiology department for head CT. There were no other complications reported.

## Discussion

The use of mechanical ventilation is usually accompanied by the need for sedative and/or analgesic agents [5-7]. Bair and colleagues reported that 85% of mechanically ventilated patients require sedatives and/or analgesics [8]. Prolonged sedation or delayed awakening is a common complication of sedation used to facilitate mechanical ventilation and is associated with increased morbidity and mortality. In addition, prolonged sedation may lead to unnecessary evaluation to exclude other causes of decreased responsiveness as neurologic examination is frequently not helpful in discriminating decreased responsiveness due to prolonged sedation from other causes. Our results suggest that head CT is of little value in patients who remain unresponsive after discontinuation of sedation in mechanically ventilated patients who have no underlying neurologic conditions and a non-focal neurologic exam. In addition to having little diagnostic value, the findings in head CT did not alter the management of these patients. Furthermore, our results suggest that head CT was performed early, within 2 days after discontinuation of sedation in majority (80%) of patients.

Administration of high dose, multiple medications and continuous infusions (vs. intermittent administration) is associated with increased likelihood of adverse reactions, prolonged sedation and mechanical ventilation, delayed weaning and increased length of stay in ICU [9-11]. In a prospective, randomized, controlled trial, Kress and colleagues reported that daily interruption of sedation until patients were awake led to a reduction in the duration of mechanical ventilation and ICU length of stay by 2.4 days and 3.5 days, respectively [1]. Furthermore, they reported a 50% reduction in the need to perform diagnostic testing to evaluate mental status in the daily interruption group (6 out 68 or 9% had head CT) than in the control group (13 out of 60 (22%) had head CT). In the control group, two brain MRI and 1 lumbar puncture were performed in addition to head CT. Our results support these findings by Kress et al. as a large majority of patients who underwent diagnostic work-up for continued unresponsiveness after discontinuation of sedation did not receive daily interruption of sedation and in contrast, the patients who did not have difficulty with awakening after discontinuation of sedation had a higher use of daily interruption of sedation. Overall only 38 of 102 (37%) patients had daily interruption of sedation, a relatively low number given the clear benefits of this strategy. To our knowledge, our data is the first to report on the usage of this strategy outside of clinical trial setting. Reasons for the low uptake are not clear but mirror those reported for the adaptation of the low tidal volume strategy for mechanical ventilation in ARDS [12,13].

In the same study, Kress and colleagues have reported only 4 of the 16 tests in the control group and 3 of the 6 tests in the intervention group provided an explanation for altered mental status [1]. Compared to the findings by Kress and colleagues, our study showed a lower percentage of diagnostic head CT scans. We speculate that this difference between our study and Kress et al. is most likely due to exclusion of patients with focal neurologic findings in our study, but it may also be due to differences in patient demographics and institutional and personal (physician-dependent) threshold for ordering a head CT for evaluation. Furthermore, the difference could also be due to higher use of daily interruption of sedation by Kress et al, which as shown by our data is associated with a lower rate of prolonged sedation, thus making a structural abnormality as the cause of unresponsiveness more likely.

Rafanan and colleagues reviewed the charts of patients who underwent head CT in all patients who were admitted to a single medical ICU [14]. Analysis of the data revealed that only the presence of a neurologic deficit predicted the results of head CT (positive vs. negative). In patients without neurologic deficit, only the presence of seizures was associated with a diagnostic CT. However, these investigators did not specifically evaluate the utility of head CT in patients who remain unresponsive after discontinuation of sedation in mechanically ventilated patients.

The transport of critically ill, mechanically ventilated patients within a hospital is challenging and may be associated with complications with increased risk of morbidity and mortality during transport [2,15,16]. Adverse outcomes including hypotension, bradycardia, tachycardia, cardiac arrest and significant hypoxemia were reported to occur in up to 70% of patients [2]. Thus, this decision should be based on a careful assessment of the potential benefits of transport weighed against the potential risk [15]. If a diagnostic testing requires a transport out of ICU and is unlikely to change the management or the outcome of the patient, the need for transport should be questioned. A review of the literature about the effect of intrahospital transport on critically ill patients showed that transport for diagnostic procedures resulted in a change in patient management only in 40–50% of cases [2]. Premature evaluation of such patients may have downsides including risks associated with patient transport and rising cost of care in the critically ill.

We acknowledge that our study had several limitations including retrospective analysis of patient charts, non-standardization of neurologic exam, and a small sample size that may preclude a meaningful interpretation of the data.

## Conclusion

We conclude that head CT is of limited diagnostic utility in mechanically ventilated patients who remain unresponsive with non-focal neurologic exams after discontinuation of sedative infusions and does not alter the management of any of the patients. Our results support that daily interruption of sedation may decrease the need for head CT in sedated, mechanically ventilated patients. A prospective, case control study may help to assess the diagnostic value of head CT in these patients.

## Abbreviations

CT: computed tomography; EEG: electroencephalography; ICU: intensive care unit; MRI: magnetic resonance imaging.

## Competing interests

The authors declare that they have no competing interests.

## Authors' contributions

GMM, HKD designed the research, JSB, MJ, NM, RB, HKD, EG collected data, JSB, RK, GMM analyzed data, GMM, RK, JSB, MJ wrote the manuscript and MJ did the statistical evaluation. All authors read and approved the final manuscript.

## Appendix

### Appendix 1

#### Inclusion Criteria

• Age ≥ 18 years

• Medical ICU admission

• Respiratory failure

• Mechanical ventilation for > 72 hours

• Unable to wake up after sedation was discontinued

• Non-focal neurologic exam

• Head CT obtained

#### Exclusion Criteria

• Known neurologic insult (*e.g. *stroke, intracranial hemorrhage, central nervous system infection) prior to ICU admission

• Focal finding on neurologic exam

## Pre-publication history

The pre-publication history for this paper can be accessed here:



## References

[B1] Kress JP, Pohlman AS, O'Connor MF, Hall JB (2000). Daily interruption of sedative infusions in critically ill patients undergoing mechanical ventilation. N Engl J Med.

[B2] Waydhas C (1999). Intrahospital transport of critically ill patients. Crit Care.

[B3] Ely EW, Truman B, Shintani A, Thomason JW, Wheeler AP, Gordon S, Francis J, Speroff T, Gautam S, Margolin R, Sessler CN, Dittus RS, Bernard GR (2003). Monitoring sedation status over time in ICU patients: reliability and validity of the Richmond Agitation-Sedation Scale (RASS). Jama.

[B4] Sessler CN, Gosnell MS, Grap MJ, Brophy GM, O'Neal PV, Keane KA, Tesoro EP, Elswick RK (2002). The Richmond Agitation-Sedation Scale: validity and reliability in adult intensive care unit patients. Am J Respir Crit Care Med.

[B5] Woods JC, Mion LC, Connor JT, Viray F, Jahan L, Huber C, McHugh R, Gonzales JP, Stoller JK, Arroliga AC (2004). Severe agitation among ventilated medical intensive care unit patients: frequency, characteristics and outcomes. Intensive Care Med.

[B6] Ely EW, Inouye SK, Bernard GR, Gordon S, Francis J, May L, Truman B, Speroff T, Gautam S, Margolin R, Hart RP, Dittus R (2001). Delirium in mechanically ventilated patients: validity and reliability of the confusion assessment method for the intensive care unit (CAM-ICU). Jama.

[B7] Ostermann ME, Keenan SP, Seiferling RA, Sibbald WJ (2000). Sedation in the intensive care unit: a systematic review. Jama.

[B8] Bair N, Bobek MB, Hoffman-Hogg L, Mion LC, Slomka J, Arroliga AC (2000). Introduction of sedative, analgesic, and neuromuscular blocking agent guidelines in a medical intensive care unit: physician and nurse adherence. Crit Care Med.

[B9] Jacobi J, Fraser GL, Coursin DB, Riker RR, Fontaine D, Wittbrodt ET, Chalfin DB, Masica MF, Bjerke HS, Coplin WM, Crippen DW, Fuchs BD, Kelleher RM, Marik PE, Nasraway SA, Murray MJ, Peruzzi WT, Lumb PD (2002). Clinical practice guidelines for the sustained use of sedatives and analgesics in the critically ill adult. Crit Care Med.

[B10] Kollef MH, Levy NT, Ahrens TS, Schaiff R, Prentice D, Sherman G (1998). The use of continuous i.v. sedation is associated with prolongation of mechanical ventilation. Chest.

[B11] Murray MJ, Cowen J, DeBlock H, Erstad B, Gray AW, Tescher AN, McGee WT, Prielipp RC, Susla G, Jacobi J, Nasraway SA, Lumb PD (2002). Clinical practice guidelines for sustained neuromuscular blockade in the adult critically ill patient. Crit Care Med.

[B12] Kalhan R, Mikkelsen M, Dedhiya P, Christie J, Gaughan C, Lanken PN, Finkel B, Gallop R, Fuchs BD (2006). Underuse of lung protective ventilation: analysis of potential factors to explain physician behavior. Crit Care Med.

[B13] (2000). Ventilation with lower tidal volumes as compared with traditional tidal volumes for acute lung injury and the acute respiratory distress syndrome. The Acute Respiratory Distress Syndrome Network. N Engl J Med.

[B14] Rafanan AL, Kakulavar P, Perl J, Andrefsky JC, Nelson DR, Arroliga AC (2000). Head computed tomography in medical intensive care unit patients: clinical indications. Crit Care Med.

[B15] Warren J, Fromm RE, Orr RA, Rotello LC, Horst HM (2004). Guidelines for the inter- and intrahospital transport of critically ill patients. Crit Care Med.

[B16] Braman SS, Dunn SM, Amico CA, Millman RP (1987). Complications of intrahospital transport in critically ill patients. Ann Intern Med.

